# Collaborative Care: Models for Treatment of Patients with Complex Medical-Psychiatric Conditions

**DOI:** 10.1007/s11920-014-0506-4

**Published:** 2014-09-14

**Authors:** Gabriel O. Ivbijaro, Yaccub Enum, Anwar Ali Khan, Simon Sai-Kei Lam, Andrei Gabzdyl

**Affiliations:** 1 NOVA University, Lisbon, Portugal; 2 The Wood Street Medical Centre, 6 Linford Road, Walthamstow, London, E17 3LA UK; 3 Public Health Department, Waltham Forest Town Hall, Forest Road, London, UK; 4 North-East London RCGP Faculty, London, UK; 5 Churchill Medical Centre, Chingford, London, UK; 6 Barking and Dagenham, Havering and Redbridge CCG’s, London, UK; 7 Wood Street Medical Centre, Walthamstow, London, UK

**Keywords:** Collaborative care, Integrated care, Long-term medical conditions (LTC), Depression, Co-morbidity, Complexity

## Abstract

Patients with co-morbidity and multi-morbidity have worse outcomes and greater healthcare needs. Co-morbid depression and other long-term conditions present health services with challenges in delivering effective care for patients. We provide some recent evidence from the literature to support the need for collaborative care, illustrated by practical examples of how to deliver a collaborative/integrated care continuum by presenting data collected between 2011 and 2012 from a London Borough clinical improvement programme that compared co-morbid diagnosis of depression and other long-term conditions and Accident and Emergency use. We have provided some practical steps for developing collaborative care within primary care and suggest that primary care family practices should adopt closer collaboration with other services in order to improve clinical outcomes and cost-effectiveness.

## Introduction

With a growing population of people living with long-term conditions, many with more than one long-term condition at a time, the answer to managing complexity and multi-morbidity will not come from doing more of the same but from changing the paradigm and finding new ways of working using a person-centred approach [1•], and collaborative care provides such an opportunity.

With the improvements in science, technology and social care as well as better environments, more people are living longer, and the trend towards an ever older population is universal in low-, medium- and high-income countries. In 2011 life expectancy had already exceeded 75 years in 57 countries of the world and, by 2017, the population aged over 65 worldwide will outnumber children under 5 [2]. This change in life expectancy, although welcome, brings its own specific challenges, which include the management of frailty, prevention of social isolation and loneliness, and management of co-morbidity. Complexity and co-morbidity will increasingly become the norm in all regions of the world. Those who commission or provide services for mental health will therefore need to think about new ways of working beyond the traditional boundaries and collaborative care to provide a cost-effective method of innovation and achievement of better health especially during the time of recession.

The recent review of projections of global mortality and burden of disease shows that NCDs (non-communicable diseases) and mental illness will continue to be the leading causes of mortality and morbidity in low-medium- and high-income countries [3•] and NCD and mental health co-morbidity has an additive effect [4–6]. In a review of 23 low- and medium-income countries, it has been estimated that US$84 billion of economic production could be lost if nothing is done to address long-term conditions in developing countries [7] and costs to the health system are significant [8••]. This reinforces the need for a collaborative approach to care.

The Global Mental Health Action Plan [9•] and the continuing movement to Universal Health Coverage (UHC) [10] provide a challenge for all actors in health care provision and delivery who strive to deliver evidence-based health care with a good outcome in the face of increasing global morbidity, ageing and the development of new medical technologies within the context of ever dwindling resources, recognising that mental and physical health co-morbidity will continue to be the norm for many and not the exception.

The WHO Mental Health Action Plan 2013-2020 has provided a platform from which all partners can work together towards enabling individuals to achieve full health. This article provides some evidence to support the need for collaborative care and provides some practical examples of how to deliver a collaborative/integrated care continuum.

## Collaborative Care Versus Integrated Care

Social determinants play an important part in health care outcomes and health care delivery, and successful integration/collaboration must go beyond the health sector because people with mental health issues have needs that go beyond health and social care [11]. There is the need to integrate mental and physical health care delivery, social care including housing provision, education, physical health promotion, mental health promotion, mental health advocacy and spirituality across the life course. This will need to be supported by interdisciplinary and multidisciplinary training of the workforce including the creation of a new cadre of family doctors with enhanced skills such as the Primary Care Practitioner with a Special Interest (PwSI) [12].

Although Collaborative Care and Integrated Care are terms that have been used internationally to describe a model of care specifically designed to improve mental health care within a primary care setting, these terms are not used consistently [13•, 14•].

Collaborative care is an effective model for integrating behavioural (mental) health care into primary care medical settings. It aims to improve the physical and mental health of people with mental illness. It specifically aims to develop closer working relationships between primary care (family doctors or GPs and practice nurses) and specialist health care (such as Community Mental Health Teams) [15••].

Stroshal [16] defined Collaborative Care as ‘Behavioural Health (mental health) working with Primary Care and defined Integrated Care as Behavioural Health (mental health) working within, and as part of, Primary Care Team’.

**Fig. 1:**
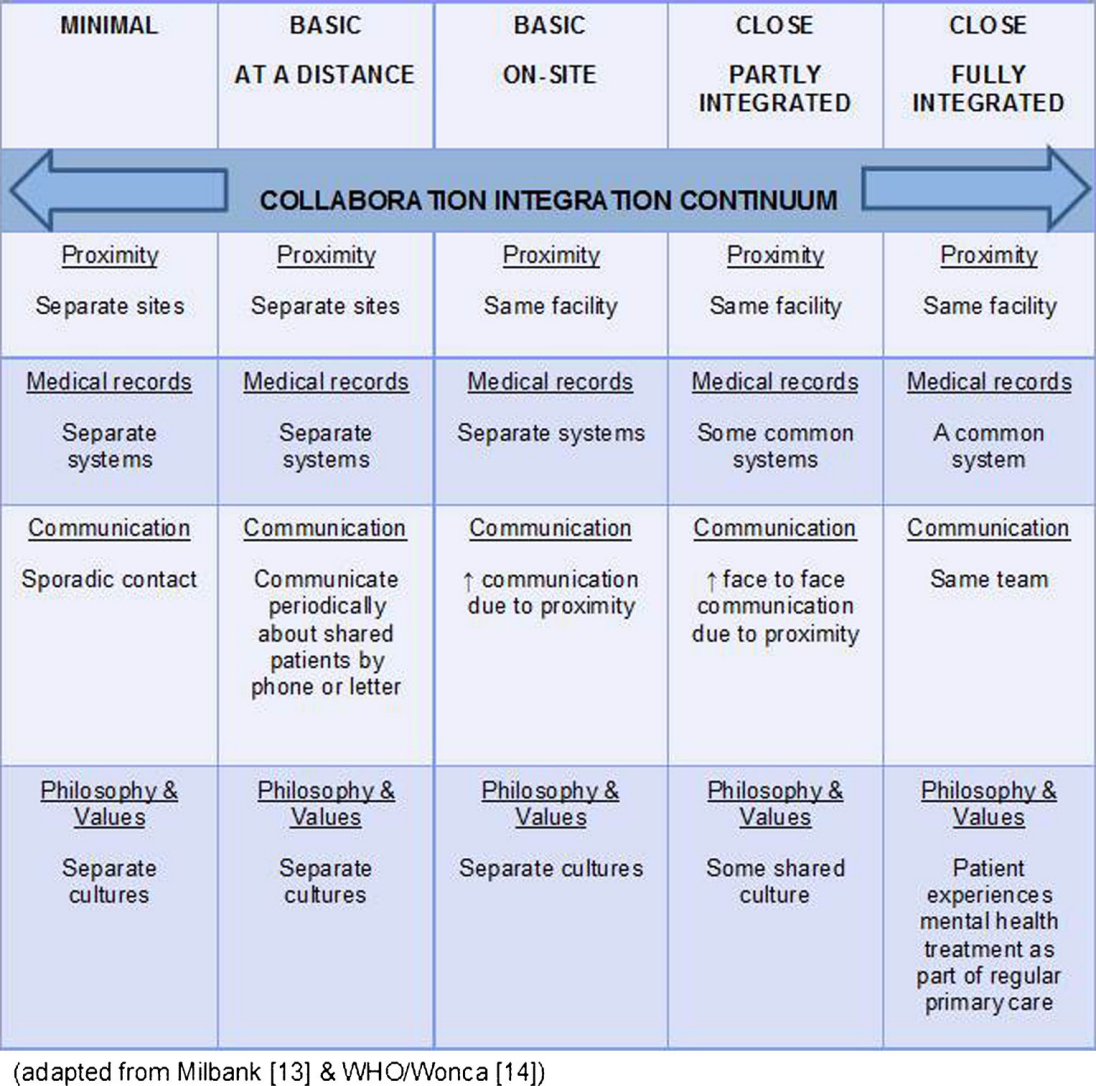
Conceptualising the collaboration/integration continuum

A functional and practical way to conceptualise the relationship is as a continuum (see Fig. 1). At one end of the continuum there is minimal integration, with mental health services delivered separately from Primary Care (different locations, separate care records and sporadic contact between the agencies). At the other end there is full integration (same team providing both Primary Care and mental health services at the same location with a common care records system).

When this model is applied to many current clinical systems, many primary care practices would be classed as Minimal or Basic collaboration, yet the essence of adopting the Collaborative and Integrated care continuum is to achieve best practice as close to full integration as possible. Full integration, which is difficult to achieve, requires practices to use the same facility, share a common records management system and supporting IT, work in the same team or network, and work toward the same values, philosophy and principles.

Close collaboration/integration will deliver a biopsychosocial model of care by recognising that there is a need to bring together a skill mix to best support the individual experiencing mental illness, recognising that many people with mental illness have other co-morbidities that may be social, psychological, psychiatric and physical [16].

Access to health and social care is often chaotic for people with the complexity and co-morbidity associated with mental illness because there are multiple access points and challenges to overcome [17] before appropriate care can be delivered. To achieve this, services need to be well co-ordinated.

## Collaborative Care – The Evidence

There have been many reviews of collaborative care for the management of patients with chronic illnesses. A randomised controlled trial in 14 primary care clinics in an integrated health care system in Washington State studied patients with depression and poorly controlled diabetes, coronary heart disease or both and concluded that, compared with care as usual, collaborative care involving nurses led to significant improvement in the management of depression and chronic diseases [18•]. In addition to clinical effectiveness, collaborative care has also been demonstrated to be cost-effective [19••].

A meta-analysis of collaborative care for depression and diabetes mellitus noted that patients with diabetes and depression are poorly managed in primary care, which is associated with delayed diagnosis of depression and, once recognised, poor treatment outcomes. Adopting a collaborative approach led to better depression outcomes and improved adherence to treatment for depression and diabetes, suggesting that there is a need to focus on collaborative care that emphasises improvement of the concurrent management of both conditions [20••].

Another meta-analysis of practice-based interventions for depression concomitant with a range of chronic medical conditions concluded that collaborative care interventions improved outcomes for depression and quality of life in primary care patients with a variety of medical conditions, although the effect was less pronounced in diabetes care [21••].

The evidence for the usefulness of collaborative and integrated care for co-morbidity goes beyond common mental disorders, diabetes and coronary heart disease. For example, in respiratory medicine there is an increased risk of depression in COPD (chronic obstructive airways disease) [22], and anxiety also contributes to the use of resources and the cost of COPD management [23–25]. Dealing with COPD alone in primary care, without addressing mental illness co-morbidity, will not lead to the most effective clinical outcomes. Applying the principles of collaborative or integrated care will bring together primary care and respiratory specialists working together to deliver more effective clinical outcomes whilst delivering value for money.

Unützer et al. [26] noted there have been over 70 randomised controlled trials of collaborative care in common mental health disorders that have shown this approach to be more clinically and cost-effective. This suggests that application of the model could substantially improve physical and mental health care in the US Medicare Medicaid system and that Collaborative Care Programmes are one approach to integration and allow primary care providers, care managers and psychiatric consultants to work together to provide better care and monitor patient progress.

There should be integration in health research, policy and practice system-wide, and collaboration must go beyond the health sector. The well-being of the most vulnerable of health system users, whose mental and physical symptoms lead to disorders with persistent impairments, may be a sensitive indicator of a society’s need for integrated care and that full social participation for vulnerable groups requires sustained access to jobs, schools and other services. This requires cooperation among education, social services, labour and justice sectors [27].

Many successful models of integrated care share common principles. The Wales Health and Wellbeing Best Practice and Innovation Board [28], drawing together some evidence on the determinants of effective integration of health and social care to help inform service re-design, summarised the key determinants as:

- Clarity of strength of purpose—having a shared vision, culture and values that deliver person-centred services based on shared outcome frameworks
- Collaborative leadership at all levels, with expert change management skills and the ability to drive cross-sector working
- A culture of learning and knowledge management that seeks to support the sharing of best practice, improvement and service development across organisational and sector boundaries
- A supportive legislative/policy environment that seeks to create the environment within which integrated services can develop
- Integrated management structures, incorporating the use of joint appointments, with unified leadership and joint governance arrangements and accountability
- Trust-based interpersonal and interprofessional multidisciplinary relationships across sectors, building on the strengths and unique contribution of each partner
- Appropriate resource environments and financial models seeking to ensure collaborative financial models, including the need for pooled budgets
- Comparable information technology (IT) and information-sharing systems that facilitate ease of communication
- Unified performance management systems and common assessment frameworks
- Collaborative capabilities and capacities, with all practitioners being skilled in integrated working and management

England has been making efforts to scale up integrated/collaborative care. A recent report for the Department of Health in England covering 16 Integrated Care Pilots (ICPs) [29••], some of which specifically included some mental health and dementia services, concluded that where there had been perceived benefits, facilitators to ICP success included strong leadership and pre-existing relationships at a personal level across organisations, shared values, collective communicated vision, investment of effort in widespread staff engagement and the provision of education and training specific to service change.

Large-scale, complex integrations were a barrier to success, as were staff concerns about changes to their roles or even threat to their jobs and poor IT connectivity between systems and organisations in a holistic fashion.

This is consistent with the findings of a review by the Kings Fund in the UK [8••]. An example from New Zealand also follows the same principles to achieve success in the delivery of integrated care [30].

One of the tools that can be used to integrate across sectors and services, especially for those with long-term health conditions with multi-morbidity who may also be vulnerable, is through the use of Navigators or care co-ordinators. In the past most care co-ordinators have been nurses or other clinicians. Non-clinicians such as Navigators can also be effective in supporting improved integration/collaboration.

A Navigator is a single named individual who can help people navigate their way through complex systems across health, social care, housing, employment and education (among other services) and help to pull together integrated care packages [31, 32]. This would go a long way to ensuring that people received effective integrated care.

## Case Example – Cost and Co-Morbidity, the Experience of Waltham Forest, London

Waltham Forest is a Borough in East London with a diverse population of 258,249 comprising 42 % Black and Asian minority ethnicities. It is 51 % female with an elderly population in the north of the borough and younger population in the south. There is a high birth rate, a high prevalence of low-birth-weight babies and a relatively young population compared to England, and it is above the national average in the 0-10 and 20-44 age groups; the older population is projected to grow. Waltham Forest is the 15th most deprived borough in England, levels of deprivation are increasing over time, and 33 % of households are defined as income deprived and 20 % of households have no member in employment.

As part of Waltham Forest’s initiative to reduce costs and improve clinical outcomes a software package called Health Analytics was developed to capture patient data in general practice and secondary care. The data collected using Health Analytics specifically captured Accident and Emergency attendance, in-patient hospital admissions, out-patient attendance, general practice appointments and diagnosis of long-term health conditions. These data were then used to calculate the cost of care per 1,000 patients registered at each practice and aggregated for all individual general practice providers in Waltham Forest taking into account the total number of A&E attendance per year and their cost, the number of A&E admissions per year and their cost, the number of short stay admissions (<3 days) and their cost, and the number of longer stay admissions (>3 days) and their cost. The data were reviewed to test whether there was a relationship between the prevalence of depression and the prevalence of other long-term health conditions in the Waltham Forest general practice population.

**Fig. 2:**
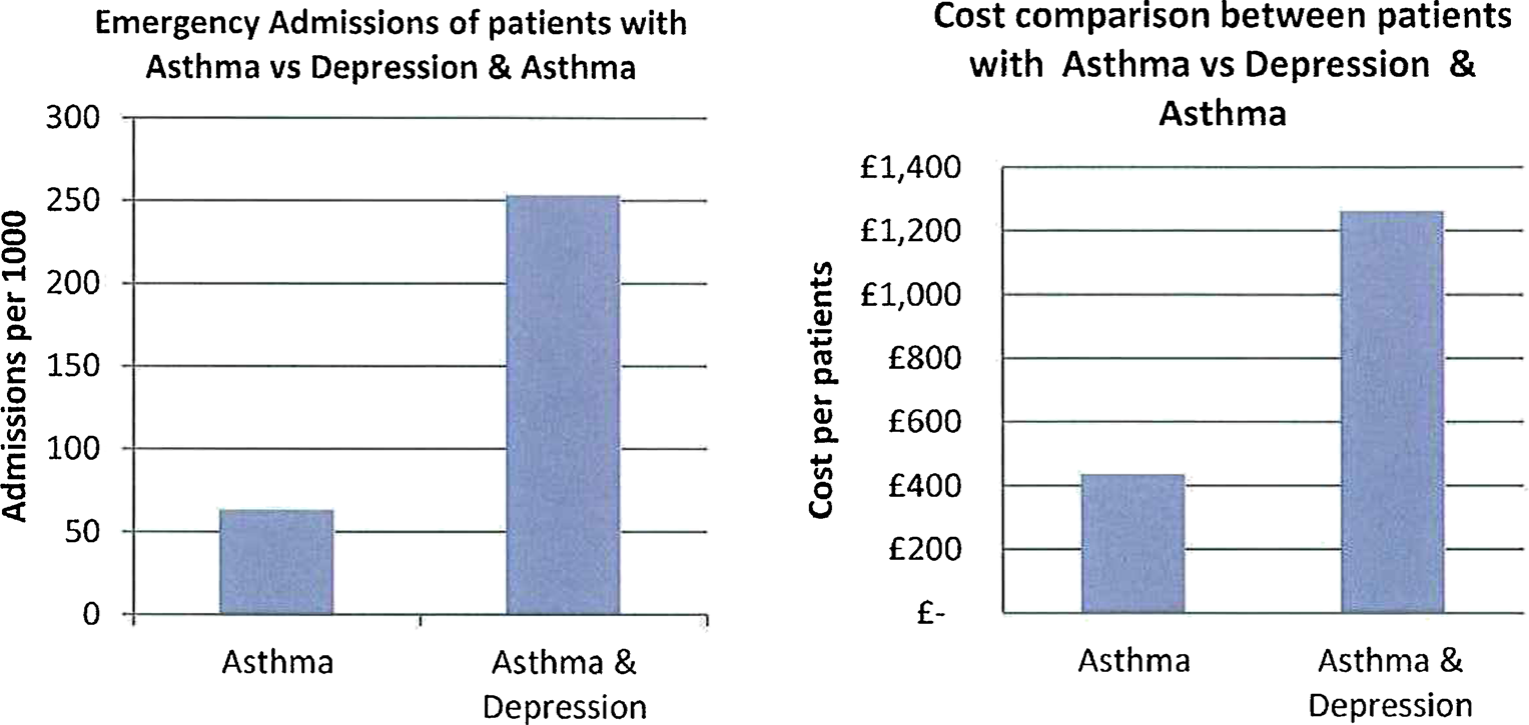
Asthma, depression, A&E admissions and cost in Waltham Forest 2011-2012

**Fig. 3:**
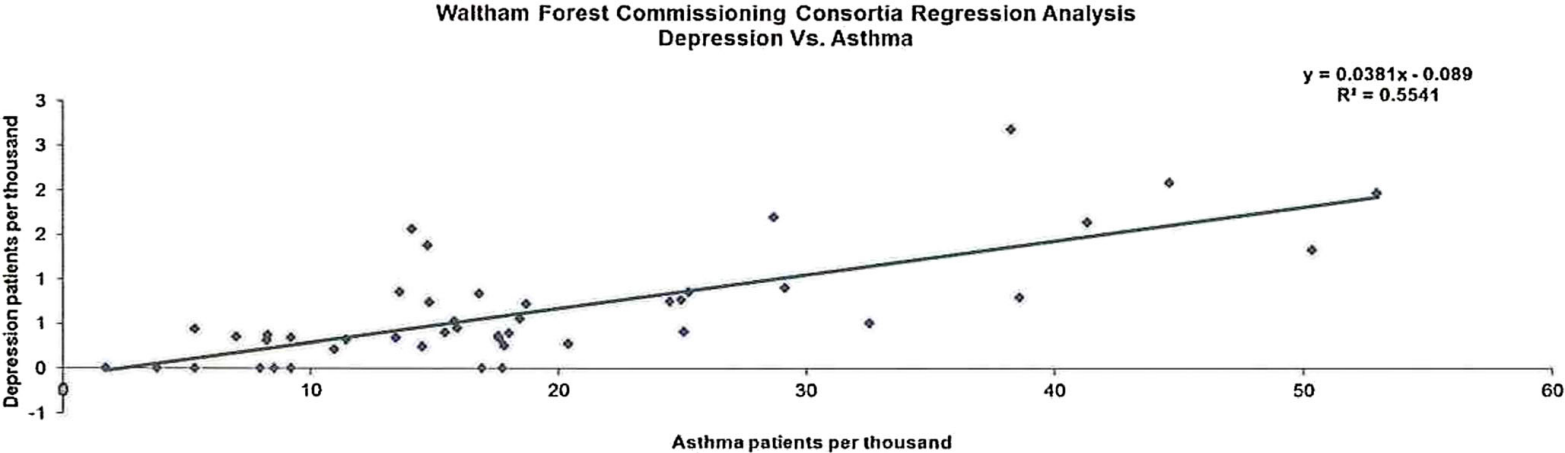
Prevalence of asthma and depression per Waltham Forest GP practice 2011-2012

**Fig. 4:**
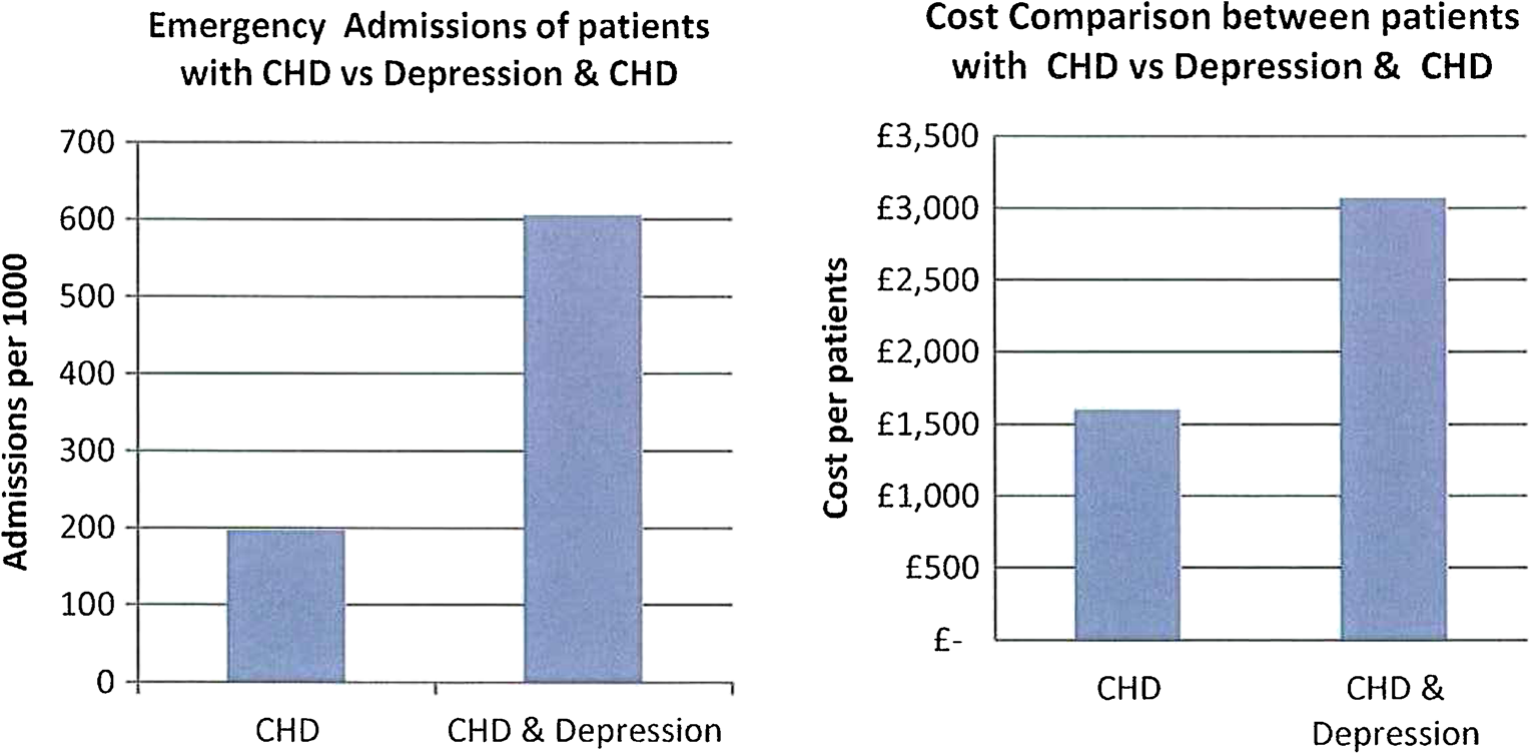
Coronary heart disease (CHD), depression, A&E admissions and cost in Waltham Forest 2011-2012

**Fig. 5:**
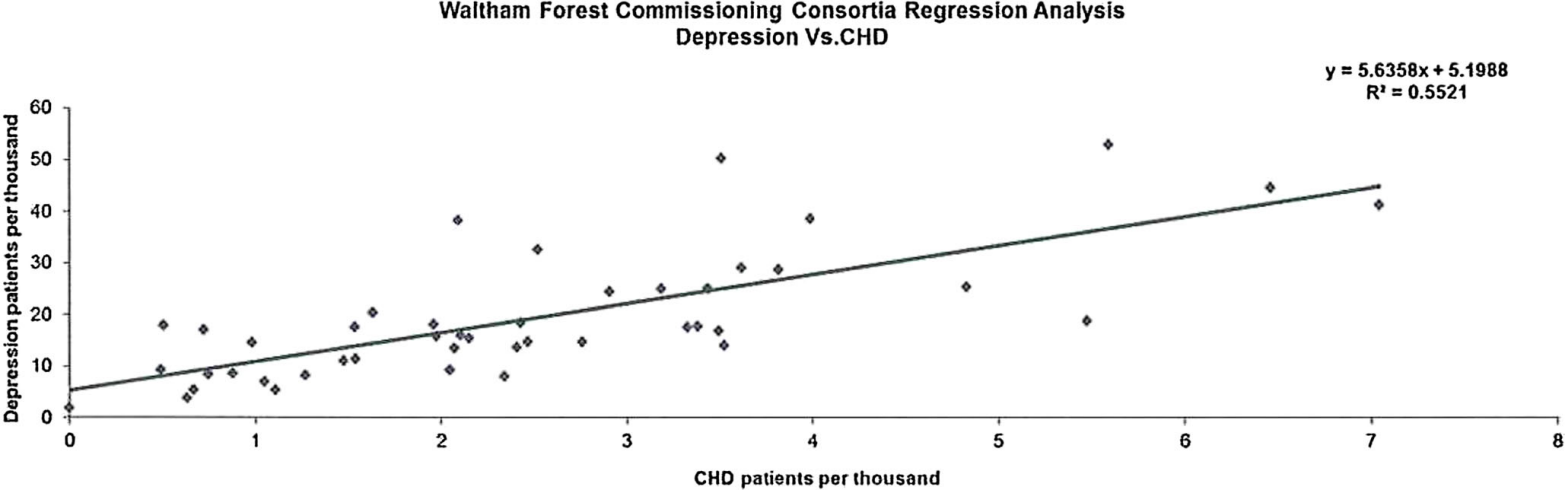
Prevalence of coronary heart disease (CHD) and depression per Waltham Forest GP practice 2011-2012

**Fig. 6:**
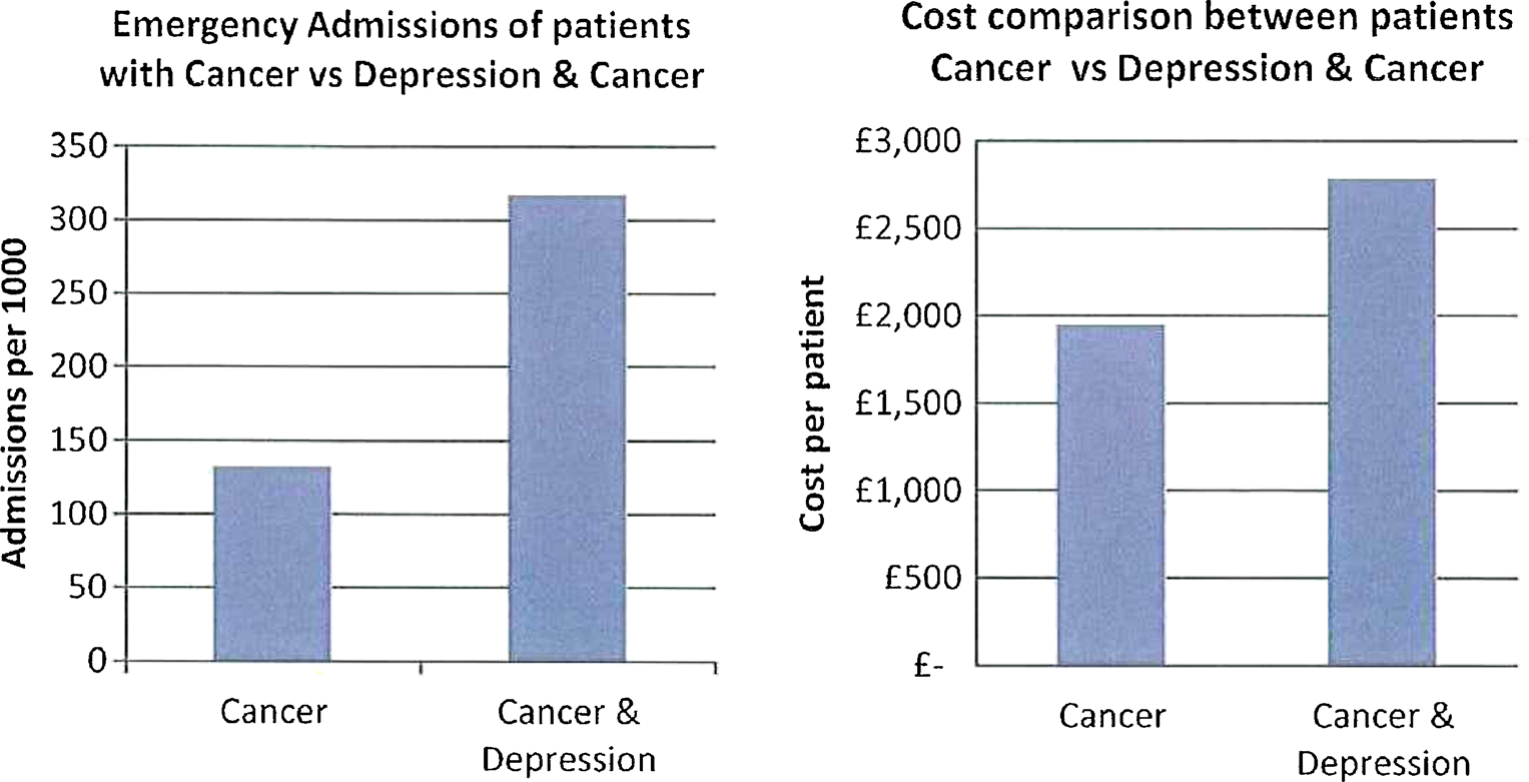
Cancer, depression, A&E admissions and cost in Waltham Forest 2011-2012

**Fig. 7:**
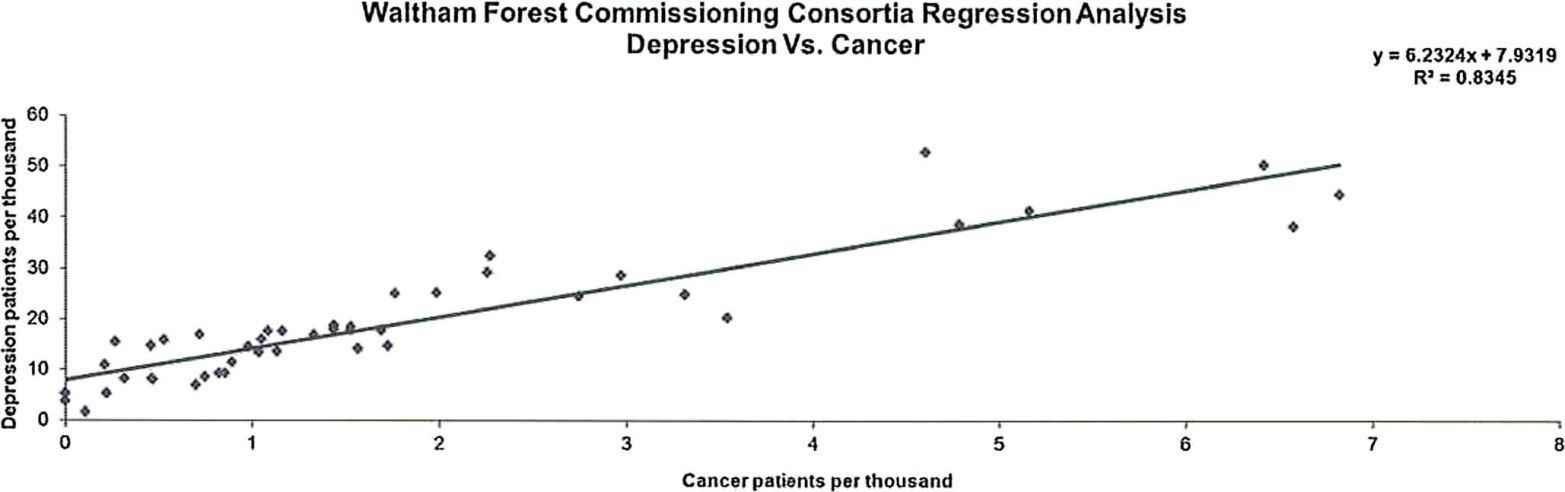
Prevalence of cancer and depression per Waltham Forest GP practice 2011-2012

**Fig. 8:**
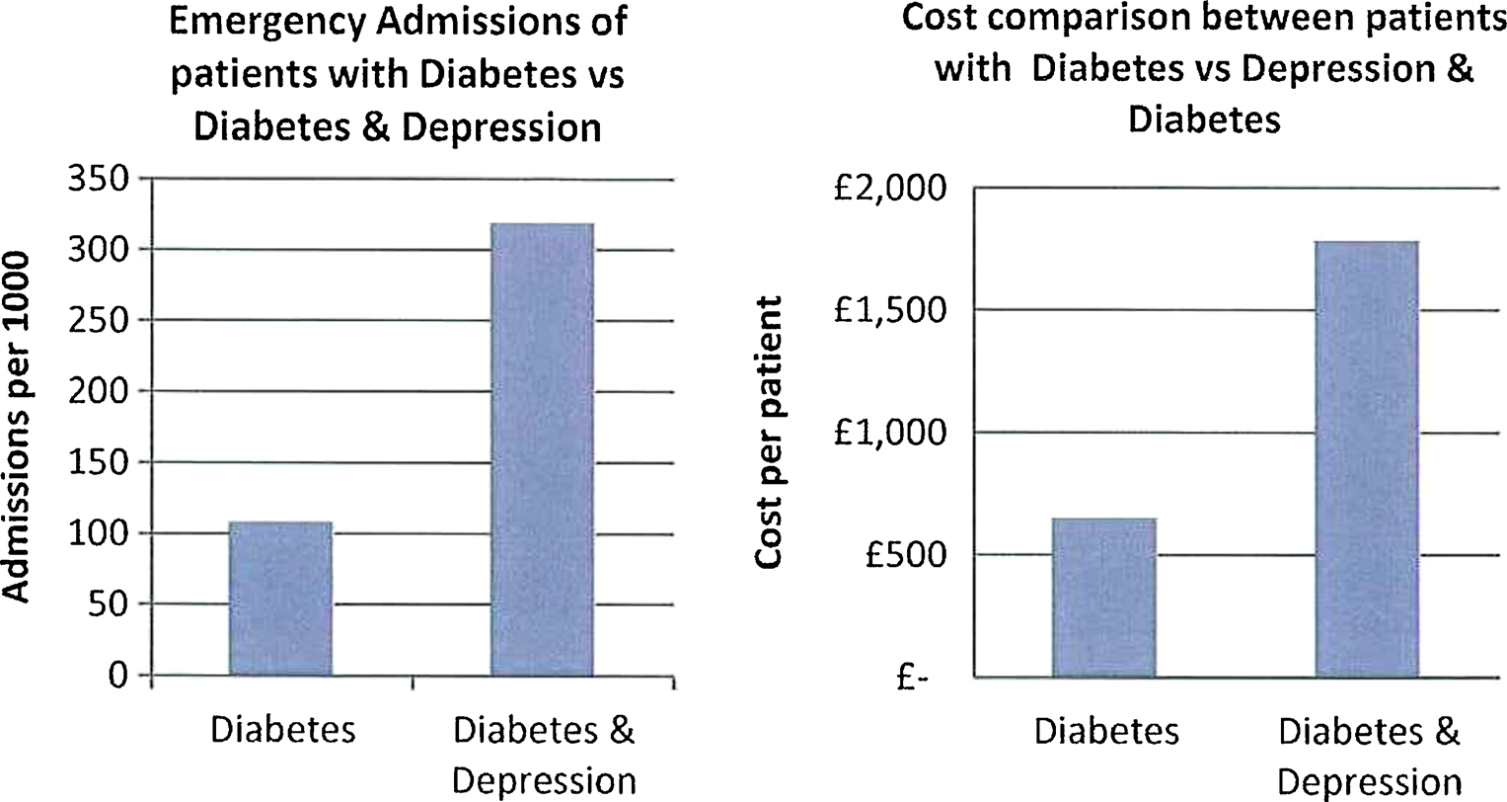
Diabetes, depression, A&E admissions and cost in Waltham Forest 2011-2012

**Fig. 9:**
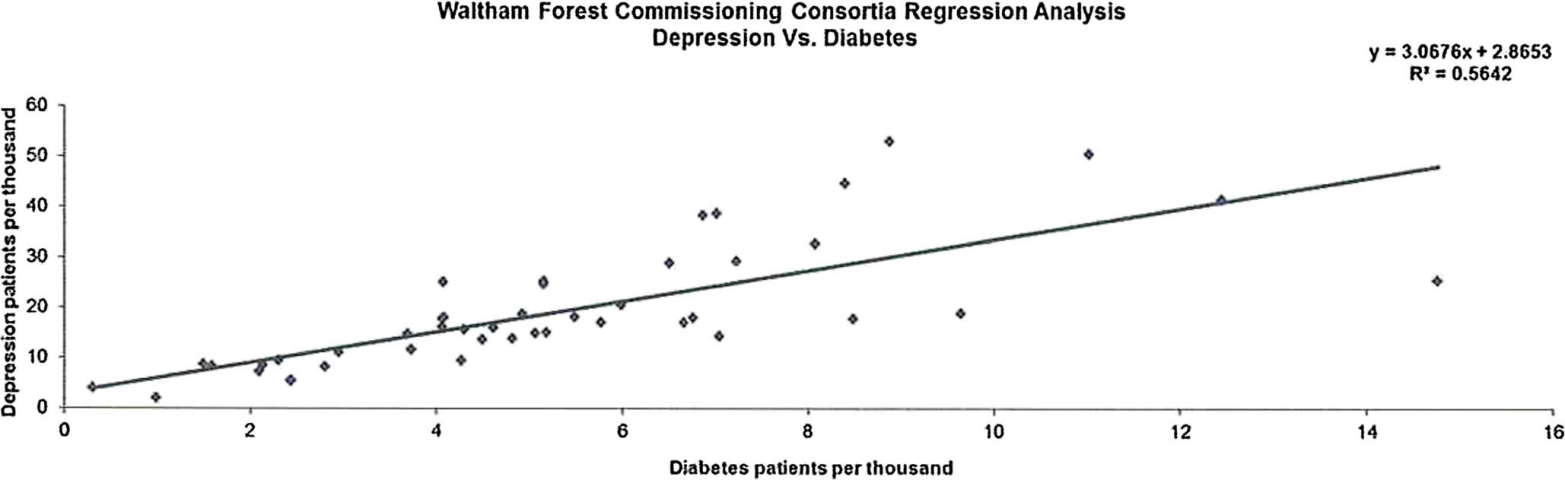
Prevalence of diabetes and depression per Waltham Forest GP practice 2011-2012

**Fig. 10:**
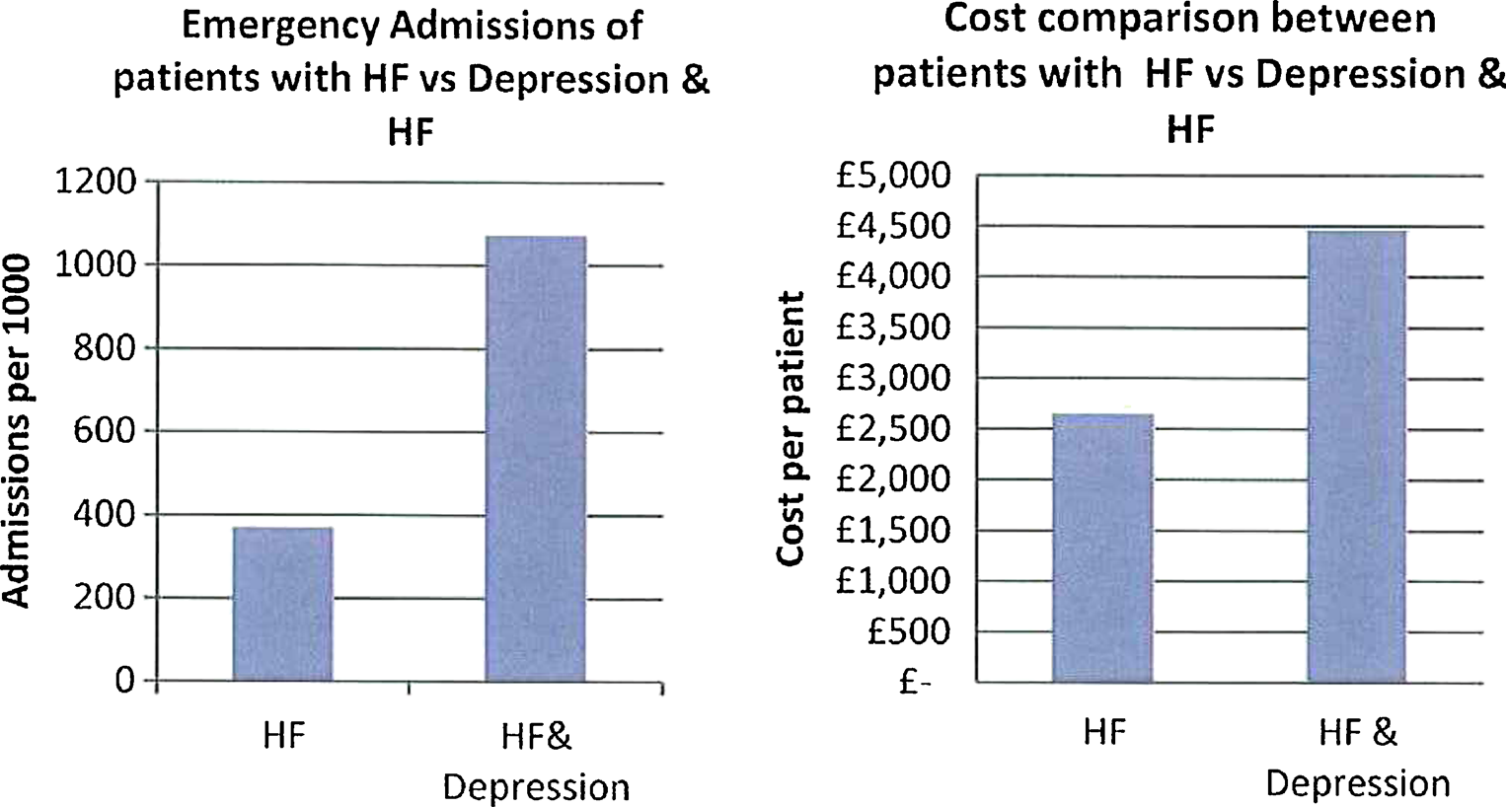
Heart failure (HF), depression, A&E admissions and cost in Waltham Forest 2011-2012

**Fig. 11:**
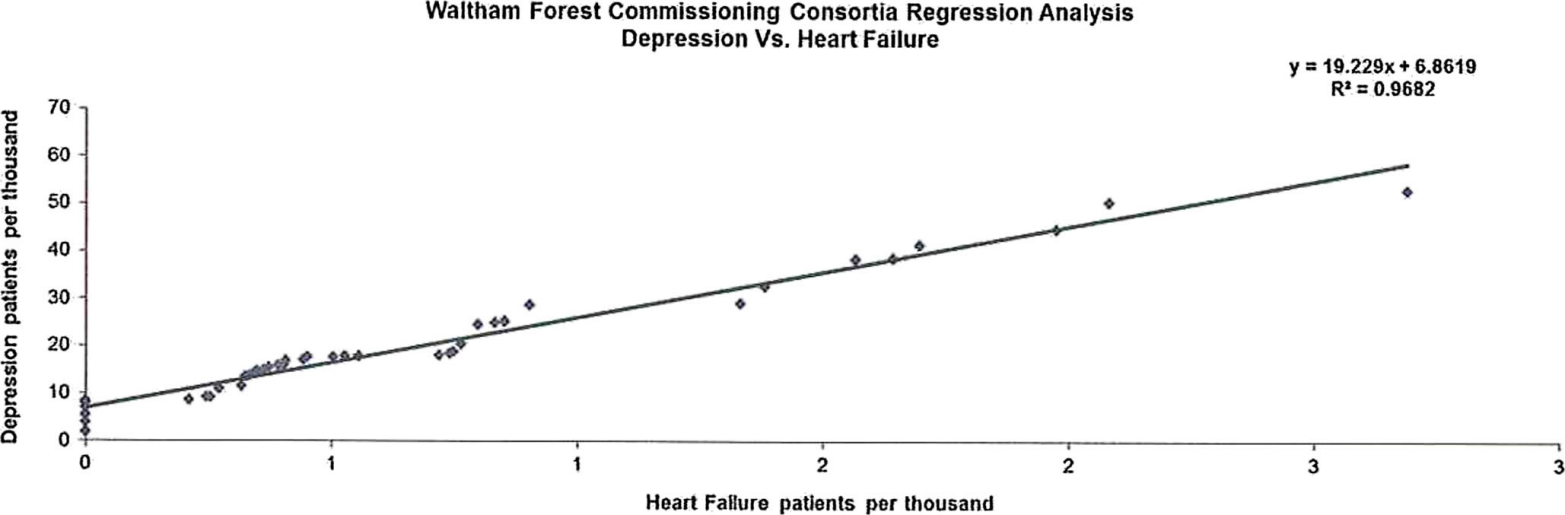
Prevalence of heart failure (HF) and depression per Waltham Forest GP practice 2011-2012

**Fig. 12:**
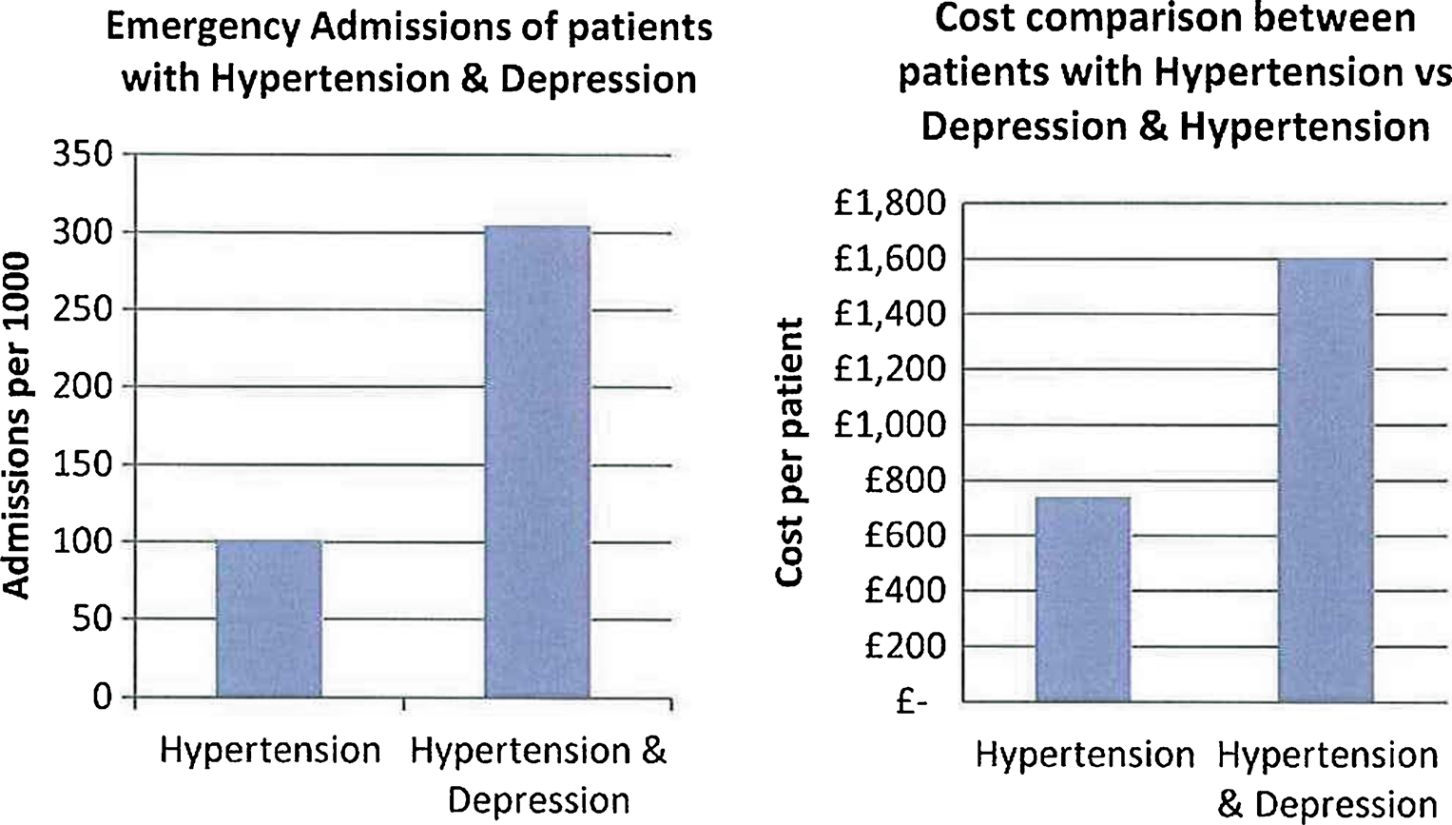
Hypertension, depression, A&E admissions and cost in Waltham Forest 2011-2012

**Fig. 13:**
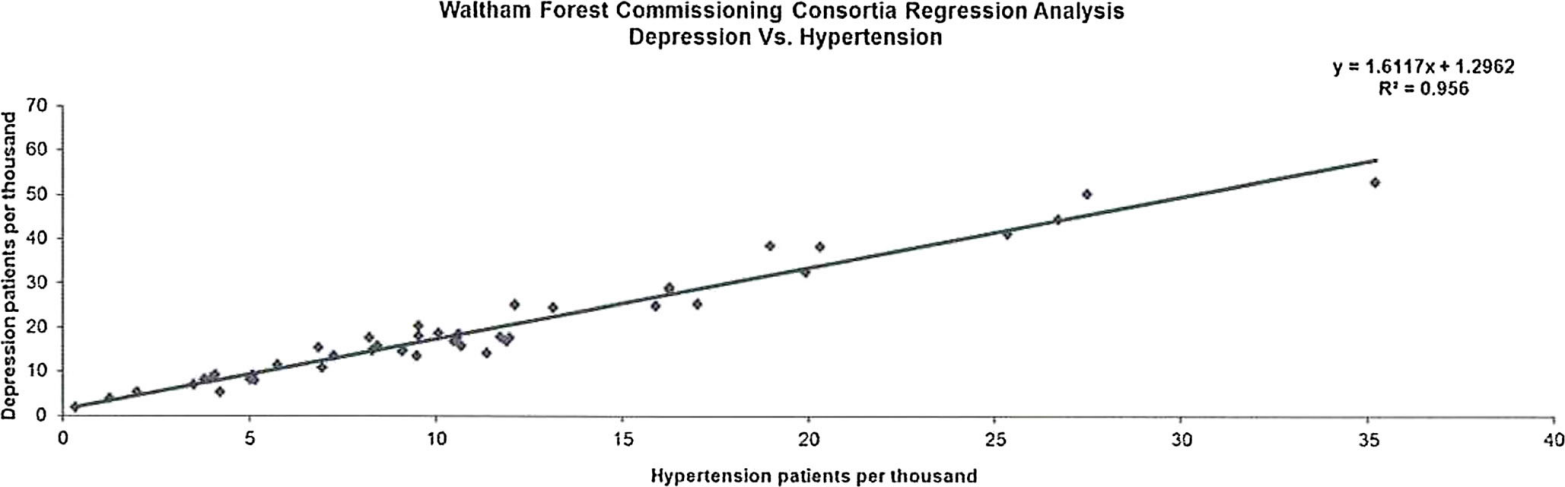
Prevalence of hypertension and depression per Waltham Forest GP practice 2011-2012

The data presented in this case study are for a total Waltham Forest patient population of 258,249 as registered in 2011-2012. Figures 2, 3, 4, 5, 6, 7, 8, 9, 10, 11, 12, and 13 show that when depression is co-morbid with other long-term health conditions, the cost of treatment is significantly increased and is also associated with a disproportionate use of accident and emergency care. This is true for all the other long-term conditions evaluated. For example, the average cost per patient with asthma and depression is almost three fold compared to the cost per patient of asthma alone. The points on each correlation represent a general practice service and for each condition the correlation graphs show that practices with high prevalence of depression also have high prevalence of depression. In Waltham Forest the association is strongest for heart failure and hypertension.

The left side of Fig. 2 compares the A&E admission rates for patients with a diagnosis of asthma alone and those with a diagnosis of asthma co-morbid with depression. The right side of Fig. 2 compares the average cost per patient for those with a diagnosis of asthma alone (£437.00) and those with a diagnosis of asthma co-morbid with depression (£1,263.00).

The points on each correlation in Fig. 3 represent a general practice service and the correlation graph shows that practices with high prevalence of depression also have high prevalence of asthma.

The left side of Fig. 4 compares the A&E admission rates for patients with a diagnosis of coronary heart disease (CHD) alone and those with a diagnosis of coronary heart disease (CHD) co-morbid with depression. The right side of Fig. 4 compares the average cost per patient for those with a diagnosis of coronary heart disease (CHD) alone (£1,603.00) and those with a diagnosis of coronary heart disease (CHD) co-morbid with depression (£3,072.00).

The points on each correlation in Fig. 5 represent a general practice service and the correlation graph shows that practices with high prevalence of depression also have high prevalence of coronary heart disease (CHD).

The left side of Fig. 6 compares the A&E admission rates for patients with a diagnosis of cancer alone and those with a diagnosis of cancer with depression. The right side of Fig. 6 compares the average cost per patient for those with a diagnosis of cancer alone (£1,950.00) and those with a diagnosis of cancer co-morbid with depression (£2,786.00).

The points on each correlation in Fig. 7 represent a general practice service and the correlation graph shows that practices with high prevalence of depression also have high prevalence of cancer.

The left side of Fig. 8 compares the A&E admission rates for patients with a diagnosis of diabetes alone and those with a diagnosis of diabetes co-morbid with depression. The right side of Fig. 8 compares the average cost per patient for those with a diagnosis of diabetes alone (£650.00) and those with a diagnosis of diabetes co-morbid with depression (£1,786.00).

The points on each correlation in Fig. 9 represent a general practice service and the correlation graph shows that practices with high prevalence of depression also have high prevalence of diabetes.

The left side of Fig. 10 compares the A&E admission rates for patients with a diagnosis of heart failure alone and those with a diagnosis of heart failure co-morbid with depression. The right side of Fig. 10 compares the average cost per patient for those with a diagnosis of heart failure alone (£2,646.00) and those with a diagnosis of heart failure co-morbid with depression (£4,460.00).

The points on each correlation in Fig. 11 represent a general practice service and the correlation graph shows that practices with high prevalence of depression also have high prevalence of heart failure.

The left side of Fig. 12 compares the A&E admission rates for patients with a diagnosis of hypertension alone and those with a diagnosis of hypertension co-morbid with depression. The right side of Fig. 12 compares the average cost per patient for those with a diagnosis of hypertension alone (£741.00) and those with a diagnosis of hypertension co-morbid with depression (£1,607.00).

The points on each correlation in Fig. 13 represent a general practice service and the correlation graph shows that practices with high prevalence of depression also have high prevalence of hypertension.

Scheduled care is less costly and more cost-effective in primary care and secondary care. Waltham Forest patients with long-term conditions co-morbid with depression showed increased use of episodic care. This case example from the London Borough of Waltham Forest illustrates the need for a collaborative approach for dealing with co-morbid long-term conditions. Depression increases the cost of the management of other long-term health conditions and consistent with the findings in the literature [4–6] effective management of both the depression and the long-term physical condition is necessary to achieve the best outcomes.

Waltham Forest is currently evaluating a range of pilots that have been initiated in response to our findings. These pilots have highlighted the need to improve the sharing of data and information between health and social care professionals and learning together to enable better understanding of each other’s work streams.

## Developing Your Integrated Service

**Fig. 14:**
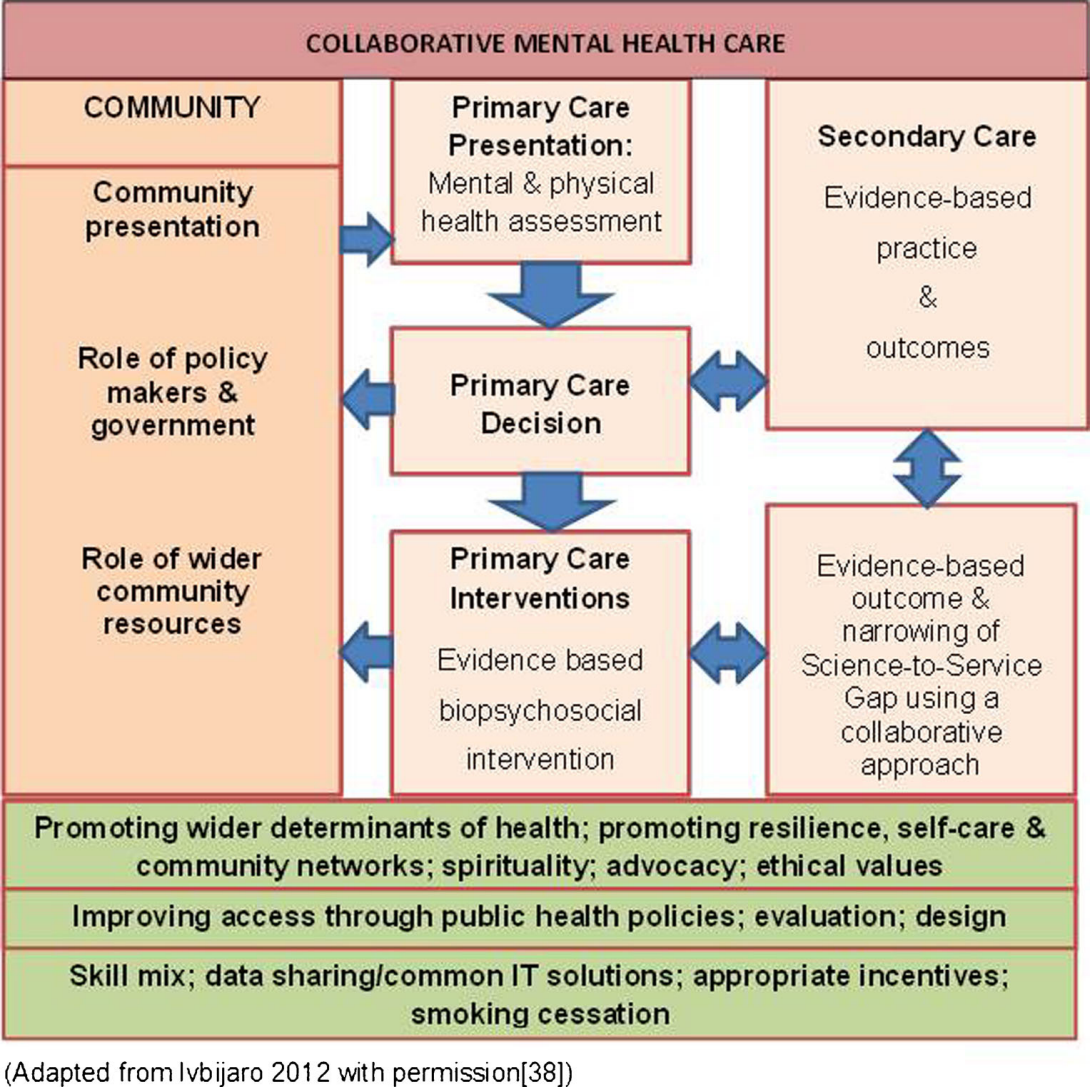
Collaborative mental health care – a schema

One of the aims of this article is to help individual practices and practitioners to implement or accelerate their collaborative/integrated approach to patient care. In order to develop an integrated/collaborative care service to suit your local population, you will first need to understand the population you are serving and the existing pathways to care. This will include an understanding of the determinants of health, the legal framework in which you practice, the range of third sector or non-government organisations available in your area and the provision delivered by existing secondary care providers. (See Fig. 14) [38].

There will need to be a business model and a project plan with clear time lines including the skill mix and workforce necessary (such as doctors, nurses, psychologists, health care assistants, etc.) to deliver the model. It is important to recognise that some of the tasks currently undertaken by clinicians can be done by others of varying grades. For example, rather than trained nurses, navigators may be able to deliver care co-ordination. You have to deal with physical and mental health conditions together, as part of a stepped care approach [33•, 34–38].

Collaborative/integrated care requires accountability from all the organisations involved in the partnership or collaboration. It requires clear clinical leadership and a methodology for information sharing. It is important to support this through a clear methodology of payments or incentives that are clearly established and understood before the project begins. This forms the foundation for effective collaboration/integration.

There are some specific tasks that need to take place in primary care. These will include training staff in patient presentation, assessment and diagnosis. Once diagnosis is made it should be entered onto the practice disease register. There is also a need to train staff to stratify patients based on the risk and seriousness of their condition so that they can be directed to the right level of resources as early as possible.

Primary care interventions need to be supported by an agreed clinical protocol and guideline, and it is often better to adopt a stepped care approach. Clinical protocols and guidelines should also include a clear statement of when to refer to secondary care and when secondary care should discharge back to primary care.

It is essential that all partners agree to the clinical protocols and guidelines for each condition so that they are universally adopted across the collaboration, underpinned by good record keeping and information that can be shared between partners. Patients, their families and carers should be part of the decision-making, and there should be continuous evaluation of outcomes so that services can be re-designed whenever the local population needs change.

Good collaborative/integrated care must have cognisance of community and personal resilience, including self-care, and should universally promote smoking cessation as we now know that smoking cessation is associated with a reduction in depression, anxiety and stress and associated with improved mood and quality of life [39••]. This is an activity that can take place across the collaborative, be it community, primary or secondary care.

## Conclusion

The management of complexity and multi-morbidity, especially of long-term conditions and depression, will continue to pose challenges for those who commission health services and for those who deliver clinical interventions. Primary care will play an increasingly significant role with the move to universal health coverage. There is a need to develop new ways of delivering clinical care to a diverse, ageing population with increasingly complex needs arising from the multi-morbidity associated with mental illness, especially depressive disorder.

This review has brought together the current best evidence supporting the collaborative care model in primary care. We have shown that when long-term medical conditions are co-morbid with depression clinical outcomes worsen and economic costs increase.

There is a growing evidence base supporting a collaborative approach to the delivery of clinical care because this is associated with better individual and community health outcomes and is also economically efficient. We have put forward a schema to illustrate how a collaborative system may be put into practice.

There is evidence that collaborative care is clinically and cost-effective in the management of care of patients with co-morbid depression and other long-term conditions. Primary care family practices should adopt closer collaboration with other services in order to improve clinical outcomes. The case example from the London Borough of Waltham Forest shows that there is a need to invest not just in physical health care but also in mental health care because the prevalence of depression increases as the prevalence of other long-term conditions increases as there is a disproportionate rise in cost when managing comorbid mental and physical health conditions.

The literature evidence illustrates that the experience in the London Borough of Waltham Forest is not unique so there is a need to encourage innovation through collaborative care because it affords the opportunity to manage co-morbidity and multimorbidity in a cost-effective way.
